# Assessing the Potential of NGF-Differentiated PC12 Cells as a Model for Synaptic Transmission

**DOI:** 10.1007/s12035-025-05562-5

**Published:** 2025-12-10

**Authors:** Grischa Ott, Jana Leuenberger, Niels Ntamati, Andrey Ivanov, Thomas Nevian, Iman Rostami, Benoît Zuber

**Affiliations:** 1 Institute of Anatomy, University of Bern, 3012 Bern, Switzerland; 2 Department of Physiology, University of Bern, 3012 Bern, Switzerland; 3 Department of Anatomy, Histology, and Embryology, Medical University-Sofia, 1431 Sofia, Bulgaria

**Keywords:** NGF-differentiated PC12 cells, Electrophysiology, Synaptic vesicle-like vesicle, Cryo electron microscopy, Synaptogenesis, Neuronal cell model

## Abstract

PC12 cells, derived from rat pheochromocytoma, are widely used to model neuronal differentiation and disease. Upon nerve growth factor (NGF) stimulation, they adopt a neuron-like phenotype, offering insights into neuronal processes. However, their fidelity in mimicking neuronal presynaptic organization has been debated, and their lack of postsynaptic organization is apparent. This study evaluates NGF-differentiated PC12 cells for up to 21 days post-induction, focusing on synaptic vesicle-like vesicle (SVLV) recycling. Using immunofluorescence, correlative light and electron microscopy (CLEM), cryo-electron tomography, and electrophysiology, we assessed presynaptic-like architecture and functionality. By combining ultrastructural and functional modalities, we provide a more integrated evaluation of synaptogenic features in this widely used model. We found that NGF-differentiated PC12 cells exhibit neuron-like features, including electrical excitability, neurite outgrowth, presynaptic-like ultrastructure, and protein clustering (synaptophysin, Rab3a). Additionally, their neurites develop characteristics resembling both axons and dendrites. However, they do not develop synapses. Although endocytosis was observed in neurites, exocytosis of recently endocytosed vesicles was absent, indicating a lack of spatiotemporally coupled SVLV recycling. Our findings suggest that NGF-induced neuronal differentiation of PC12 cells is limited. The cells lack the fully developed presynaptic organization required for sustained synaptic vesicle recycling. Thus, synaptogenesis is incomplete. These findings define the structural and functional boundaries of PC12 cells as a synaptic model. The limited synaptic competence of NGF-differentiated PC12 cells underscores the importance of model selection in synaptic research.

**Graphical Abstract:**

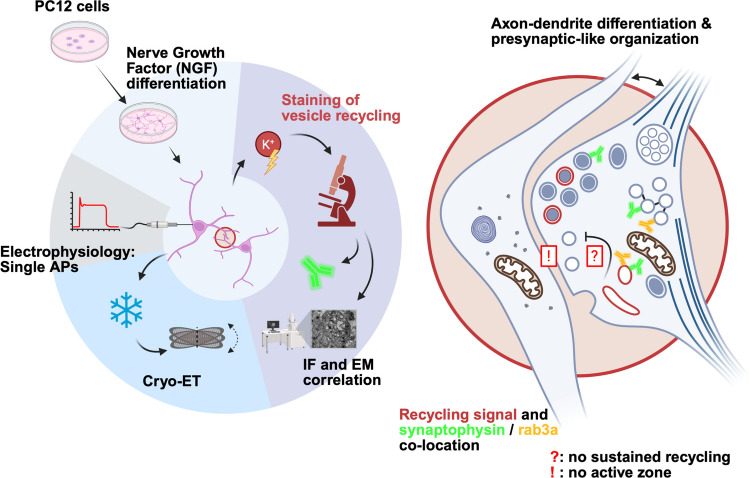

**Supplementary Information:**

The online version contains supplementary material available at 10.1007/s12035-025-05562-5.

## Introduction

Neurological and neurodegenerative disorders not only present a diverse range of clinical manifestations, but often also exhibit a highly complex pathogenesis. Understanding these disorders requires robust cell models, alongside other approaches, for unraveling the underlying neuronal machinery, identifying key defects, and exploring potential therapeutic interventions. The immortalized PC12 clonal cell line, derived from rat pheochromocytoma cells [1], has been instrumental in neurobiological research, particularly in elucidating principles of neuronal differentiation as well as the dynamics and machinery of synaptic and large dense core vesicles (LDCV) [2–7]. Its utility has extended to modeling neurotoxic effects [8–10], neuronal hypoxia (summarized in [11]) and neurodegenerative disease [12–17].

In many studies, PC12 cells have been used in their undifferentiated state, which mainly reflects their chromaffin cell origin. PC12 cells can be differentiated using NGF stimulation, by which they reversibly develop a sympathetic neuron-like phenotype, characterized by halted proliferation, neurite outgrowth, and increased synthesis of clustered synaptic vesicle-like vesicles (SVLV) containing acetylcholine (ACh), alongside electrical excitability and neurotransmitter release upon stimulation [18–20]. The resulting catecholaminergic-cholinergic phenotype reflects the role of NGF in sympathetic neuron and basal forebrain cholinergic neuron development [18, 21]. Hence, PC12 have been considered a model for neurons and neurogenesis. Relative ease of use and fast differentiation protocols further set it apart from more difficult-to-handle cell models, such as primary neuronal cultures.

Despite this, previous research has identified several limitations [11, 12], foremost the lack of proper synapses [22]—though synaptogenesis in co-culture with neurons has been described [23, 24]. We aimed to evaluate some of these constraints experimentally, focusing on whether prolonged NGF-induced differentiation enhances PC12 cell neuronal resemblance. This was informed by the fact that other neuronal models such as SH-SY5Y cells, NT2 cells, or neurons derived from induced pluripotent stem cells are typically differentiated for longer periods to achieve maturity [25–27]. Specifically, we examined the extent to which NGF-differentiated PC12 cells develop neuronal characteristics in terms of function and architecture. We investigated their ability to recycle SVLVs in a neuron-like manner, as this process is both an endpoint of neuronal differentiation and likely involved in synaptogenesis [28, 29]. We further employed conventional electron microscopy (EM) and cryo-electron tomography (cryo-ET), correlative light and electron microscopy (CLEM), and immunostaining to evaluate the cell capacity for presynaptic-like organization and formation of intercellular connections, which are precursors to synaptogenesis.

While most previous studies have focused on individual synaptic-like features in PC12 cells, we applied a multimodal approach combining high-resolution imaging and live-cell assays to more systematically assess the synaptic potential—and limitations—of this widely used model.

Our findings suggest that NGF-differentiated PC12 cells do not develop mature or functional synapses using a standard differentiation protocol. While the observed cell–cell contacts are considerably tighter than in undifferentiated cells and often display some one-sided presynaptic-like organization such as SVLV and organelle clustering, an active zone or a structure corresponding to a postsynaptic density is notably absent. PC12 cell electrical properties resemble those of immature neurons, displaying single action potentials upon current injection. Local vesicle recycling, as assessed by AM4-64 staining, could be observed and correlated with synaptophysin and Rab3a puncta, suggesting that the recently endocytosed vesicles possess some characteristics of synaptic vesicles (SVs). However, exocytosis of these vesicles was not observed in destaining experiments. This may indicate the limitations of NGF-induced neuronal differentiation in PC12. We discuss these findings in the context of current synaptogenesis literature.

## Materials and Methods

### Cell Culture

PC12 cells were maintained at 37 °C with 5% CO_2_ in high-glucose Dulbecco’s modified Eagle Medium (DMEM, Gibco, Thermo Fisher Scientific), supplemented with 5% horse serum (Gibco), 5% fetal bovine serum (Gibco), 4 mM GlutaMAX (Gibco), and 1% penicillin–streptomycin (Gibco). Cells were passaged at a 1:3 to 1:6 ratio every 1–2 weeks. For passaging, cells were passed through a 21-gauge needle to achieve a single-cell suspension. Culture conditions were informed especially by ref. [30] to help retain cell identity and secretory competence (as shown for the same cell stock in ref. [3]). Differentiation was induced using high-glucose DMEM containing 1% horse serum, 100 ng/ml rat nerve-growth factor β (NGF, Sigma-Aldrich), 4 mM GlutaMAX, and 1% penicillin–streptomycin. A cell suspension of 50,000 cells/ml was seeded onto culture ware, which was previously coated with collagen IV (R&D Systems)for 2 h at 37 °C and washed. Incubation continued with replacement of NGF-containing medium (2/3 volume) three times per week. Differentiation after a high-serum, non-NGF pre-culture [31] or omission of needle dissociation resulted in overgrowth, clumping, and film-like detachment due to locally high confluence. Additionally, higher cell seeding densities led to clumping and reduced total neurite length.

### Western Blot

PC12 cells were washed with Dulbecco’s Phosphate-Buffered Saline (DPBS, Sigma-Aldrich) and lysed on ice for 30 min using RIPA buffer supplemented with EDTA-free proteinase inhibitor (Sigma-Aldrich). The collected lysate was sonicated and centrifuged at 13,500g for 30 min, after which protein concentrations in the supernatant and pellet were determined using a bicinchoninic acid assay (Sigma-Aldrich). Samples were prepared in Laemmli buffer, boiled for 5 min at 95 °C, snap-frozen in liquid nitrogen and stored at −80 °C. Equal protein amounts were subjected to 10–12.5% SDS-PAGE, transferred to a nitrocellulose membrane (Merck), then incubated in primary antibodies in Tris-buffered Saline with Tween (TBST) at 4 °C overnight, followed by incubation with horseradish peroxidase (HRP)-conjugated secondary antibodies in TBST (for details see Table S1). Chemiluminescence generated by ECL reaction (Sigma-Aldrich), detected in a Fusion FX system (Vilber Lourmat) ensuring no oversaturation, was quantified with the GelAnalyzer software (by Istvan Lazar Jr.).

### Electrophysiology

PC12 cells cultured on 12 mm round glass coverslips were transferred to a chamber perfused with room-temperature artificial cerebrospinal fluid solution (composition in mM: 125 NaCl, 2.5 KCl, 25 NaHCO_3_, 1.25 NaH_2_PO_4_, 1 MgCl_2_, 2 CaCl_2_, 25 glucose) saturated with 95% O_2_ and 5% CO_2_. Whole-cell patch-clamp recordings were conducted using heat-pulled borosilicate glass pipettes (7–9 MΩ) filled with intracellular solution (composition in mM: 130 K-gluconate, 5 KCl, 10 Na-phosphocreatine, 4 Mg-ATP, 0.3 Na-GTP, 10 HEPES). Square-shaped currents of 500 ms duration were injected in a range from −60 to + 300 pA in 20 pA steps to elicit action potentials in the current-clamp configuration. To block sodium channels, the perfusion solution was supplemented with either 1–10 µM tetrodotoxin (TTX; Alomone Labs) or 300 µM lidocaine (Sigma-Aldrich). Spontaneous postsynaptic activity was assessed by voltage-clamping cells at −60 mV. Recorded signals were amplified and low-pass filtered at 5 kHz (BCV-700A, Dagan), digitized at 10 kHz (ITC-16, Instrutech), and analyzed using Igor Pro software (WaveMetrics), Excel (Microsoft Corporation), and R [32]. Input resistance was determined from the slope of the voltage–current relationship (ΔV/ΔI) at steady state within the linear range using current injections of −60, −40, and −20 pA.

### Immunofluorescence (IF)

PC12 cells cultured on µ-dishes (ibidi) or square coverslips (Menzel X1000, Fisher Scientific) were fixed in a 3% paraformaldehyde and 0.5% glutaraldehyde solution for 1 h, permeabilized with 0.3% Triton X-100 for 15 min, and blocked with 3% SureBlock (LubioScience) for 1 h. Primary antibody incubation was performed overnight at 4 °C in 3% SureBlock in PBS, followed by a 1-h room temperature incubation with secondary antibodies and DAPI (1:1000) in 3% SureBlock in PBS (see Table S1 for antibody and dilution information). µ-dishes were imaged in PBS and subsequently processed for EM, while coverslips were air-dried for 24 h at 4 °C in the dark and mounted on glass slides using Antifade mounting medium (Invitrogen ProLong Glass, Thermo Fisher Scientific).

### Live-Cell Staining

Live-cell staining of vesicle recycling was adapted from established protocols [33–35], using the fixable FM dye AM4-64 (Biotium, VWR International) and SCAS (Biotium) as a quencher to minimize post-stain washing time. A custom perfusion system with two 20-gauge needles, adapted to a µ-dish, facilitated gentle medium exchange during imaging. When cationized ferritin (Sigma-Aldrich) was used to label endocytosis, it was added in a concentration of 0.25 mg/ml (as in ref. [36]) and otherwise handled like AM4-64. Cells were washed in Tyrode’s solution (composition in mM: 124 NaCl, 5 KCl, 2 CaCl_2_, 1 MgCl_2_, 30 glucose, 25 HEPES; 310 mOsm/l, pH 7.4) and then incubated in Tyrode’s solution containing 10 µM AM4-64 dye to label the cell membranes for at least 2 min. They were then stimulated for 2 min with a depolarizing high-potassium solution (Tyrode’s with 70 mM KCl and 59 mM NaCl) containing the dye, followed by incubation in normal Tyrode’s solution with dye for a variable duration, usually with a minimum of 15 min to allow for staining of slow endocytosis [35]. Following this, the non-internalized dye was quenched by washing the cells in low-calcium Tyrode’s solution (0.2 mM CaCl_2_ and 5 mM MgCl_2_, to inhibit recycling) containing 0.5 mM SCAS for at least 5 min. Cells were then fixed in a solution of 3% paraformaldehyde and 0.5% glutaraldehyde for 1 h. After washing with PBS, the cells were imaged and then processed for CLEM or immunofluorescence. For destaining experiments, non-fixed, live-stained cells were stimulated with dye-free high-potassium solution under constant imaging using a large pinhole for high sensitivity to fluorescence changes and the minimally necessary laser intensity to minimize photobleaching.

### Room-Temperature CLEM

Room-temperature CLEM was conducted based on protocols from ref. [37, 38]. The ibidi µ-dish polymer was found to be incompatible with clean separation from Epon; therefore, we grew the cells on Aclar film (51 µm). The film was cleaned with ethanol and carbon-coated (15 nm coat) using a Safetec unit (safematic) through a laser-cut steel stencil (JLCPCB), producing a 250 µm alphanumeric grid. To prevent cell clustering on coated areas, a 2 nm carbon layer was applied without stencil. The film was affixed to µ-dishes using inert silicone glue. Following UV sterilization and collagen IV coating, PC12 cells were cultured, stained, fixed, washed, and imaged as described above. Regions of interest were recorded via grid coordinates for later relocation at the electron microscope. After imaging, the cells were refixed in 2.5% glutaraldehyde, washed three times with 0.15 M HEPES (Sigma-Aldrich), postfixed with 1% OsO4 (EMS) in 0.1 M Na-cacodylate buffer (Merck) at 4 °C for 1 h. After three further washes (same buffer), dehydration proceeded in graded ethanol (Alcosuisse; 70%, 80%, 96%, 15 min each) and three 10-min pure ethanol (Merck) steps at room temperature. Samples were incubated in 1:1 ethanol-Epon at room temperature, embedded in pure Epon (Fluka), and cured at 60 °C for 5 days. The Aclar film was peeled off, leaving the carbon grid on the Epon surface to locate regions of interest. Horizontal sections (1 µm) were cut with a UC6 ultramicrotome (Leica) and used for light microscopy-based orientation of cellular features. Ultrathin sections (70–80 nm) were then cut, mounted on 200-mesh copper grids (Agar Scientific), and stained with uranyless (EMS) and lead citrate (Leica) in an EMstain (Leica). Imaging was conducted on a Tecnai Spirit electron microscope operated at 80 kV acceleration voltage.

### Cryo-ET and Vesicle Morphology Analysis

PC12 cells were cultured on UV-sterilized, collagen IV-coated carbon gold EM grids (300 mesh with Quantifoil R 2/1 or 200 mesh with Lacey carbon film, EMS) in 6-well plates. After transfer to a manual plunge freezer, 4 µl of a 10 nm uncoated gold nanoparticles solution (Aurion) was added as fiducial markers. The grids were manually reverse blotted using Munktell 12/N filter paper and vitrified by rapid plunging into liquid ethane, then stored in liquid nitrogen. Cryo-ET data was collected at the Dubochet Center for Imaging (DCI) Bern using a Titan Krios G4 electron microscope equipped with a Falcon 4i direct electron detector and a Selectris energy filter, and the tomo5 software (Thermo Fisher Scientific). Tilt series were acquired at 300 kV, ×26,000 magnification, tilt range ± 60° in 2° steps, target defocus −10 µm and dose adjusted to 1–1.5e^−^/Å^2^. Tomograms were reconstructed IMOD [39], applying nonlinear anisotropic diffusion filtering [40] using parameters optimized for each tomogram.

LDCV ellipticity was assessed in the XY plane only due to poor axial resolution, using an approach as described in ref. [3]. Briefly, in the tomogram slice in which the vesicle’s major axis (*a*) was largest, the perpendicular minor axis (*b*) was measured, and ellipticity (*e*) was calculated as follows:

$$e= \frac{a-b}{a}$$

### Image Acquisition and Processing

Cells were imaged on a confocal Zeiss LSM 880 microscope using a 40 × oil immersion objective, or a 10 × dry objective for neurite analysis. Image analysis was performed in FIJI [41]. Correlation of fluorescence with EM images, and AM4-64 with immunofluorescence images, was conducted in Icy [42] using the ec-CLEM plugin [43]. Non-rigid transformations were applied as needed to correct for distortions during sample preparation. For CLEM, fluorescence image slices (~ 1 µm axial resolution) were correlated to EM data using prominent cellular features, such as lipid droplets and nuclear morphology. All other images are maximum-intensity projections of relevant slices.

Neurite length was measured in 10 × images stained with β-III-tubulin and DAPI using the NeurphologyJ macro [44]. The macro was modified to identify cell bodies in the DAPI channel as β-III-tubulin does not sufficiently stain cell bodies for automated detection. Fluorescence puncta were identified as regions of interest (ROIs) by thresholding with the maximum entropy method in CLAHE-adapted images that had been filtered using the difference of Gaussians, with optimized parameters consistently applied for each dye. Puncta overlap was identified by overlap of ROIs from different channels (i.e., dyes).

### Destaining Analysis

Time-lapse images were stabilized using FIJI’s Image Stabilizer (by K. Li). To identify areas of fluorescence loss, a difference image was generated by subtracting maximum intensity projections of five sequential images at 60 s post-stimulation and immediately pre-stimulation. Resulting puncta, representing changes in fluorescence intensity, were automatically detected as described above. ROIs were manually reviewed in time-lapse images to exclude changes due to lateral movement. To confirm that disappearing puncta had not moved out of the imaging plane (Z-axis), Z-stacks were compared before and after time-lapse imaging. Fluorescence intensity over time was extracted for each ROI using FIJI’s Time Series Analyzer (by J. Balaji). For a visual summary, intensity values were normalized to the mean of the five measurements preceding stimulation, yielding a comparable baseline at the time of high-potassium depolarization.

### Quantification and Statistical Analysis

Quantitative data from western blot and image analyses were compiled in Excel (Microsoft Corporation) and analyzed in R [32] using the tidyverse package [45]. Unless otherwise stated, results are presented as mean ± standard error (SE). Statistical tests used are indicated in the text and were applied according to the presence or absence of normality as assessed by the Shapiro-Wilks test as well as sample size. Statistical significance was assumed at *p* < 0.05.

In destaining experiments, fluorescence decay rates before and after high-potassium stimulation (i.e., reactivity to depolarization) were analyzed using a piecewise linear mixed-effects model, with slopes before and after stimulation as fixed effects and intercepts and slopes per experimental unit (punctum) as random effects. To ensure efficient modeling while maintaining interpretability, analysis was restricted to a 2-min window centered around KCl addition, during which decay approximated linearity. Models were fitted using the lme function (nlme R package [46]) with restricted maximum likelihood estimation (REML). The difference in pre- and post-stimulation slopes was evaluated via a contrast-based Z-test, with a two-sided *p*-value derived from the standard normal distribution.

## Results

### PC12 Neurite Development and Synaptic Protein Expression During NGF Differentiation

**Fig. 1:**
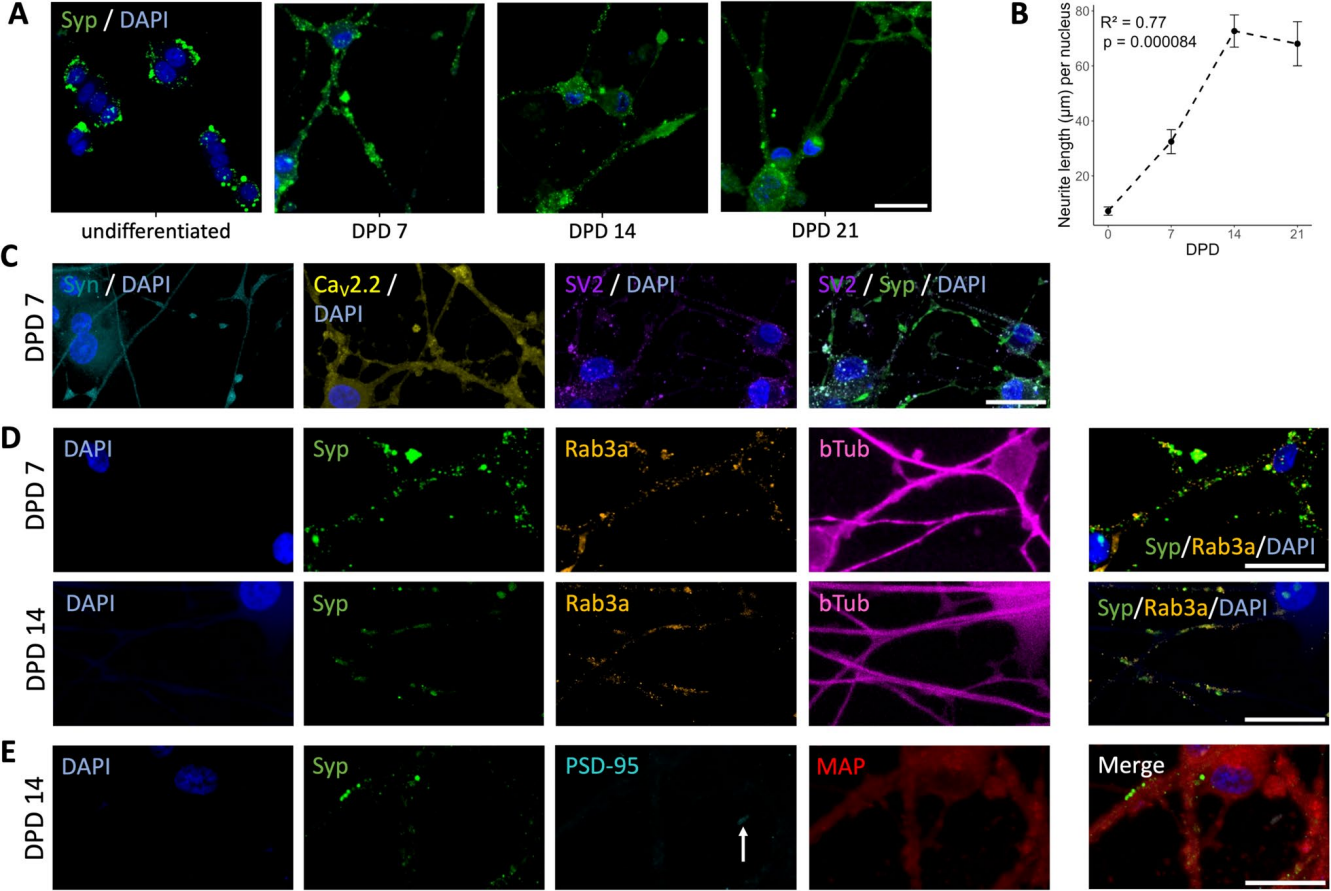
Structural characteristics during NGF-differentiation. **A** PC12 cells immunostained for synaptophysin and counterstained with DAPI show synaptophysin puncta at the cell body periphery and in neurites at all DPD. **B** Measured neurite length per cell as a function of DPD; means and SE of *n* = 3 independent differentiations per DPD. Inset: Correlation coefficient *R*^2^ of a linear regression model and associated *p*-value (< 0.05). **C** Presynaptic markers in immunostaining show increased concentrations at branching points and neurite swellings (synapsin I and Ca_V_2.2) or form puncta (SV2), some of which overlap with synaptophysin (right, overlap is intensely cyan-hued). **D** Immunostaining (at DPD 7 and 14, respectively) shows overlap of Rab3a and synaptophysin. **E** PSD-95 immunostaining shows a lack of signal except in some debris (arrow). Scale bars: 25 µm. Abbreviations: DAPI, 4′,6-diamidino-2-phenylindole; MAP-2, microtubule-associated protein 2; PSD-95, postsynaptic density protein 95; syn, synapsin I; syp, synaptophysin; bTub, β-III-tubulin

**Fig. 2:**
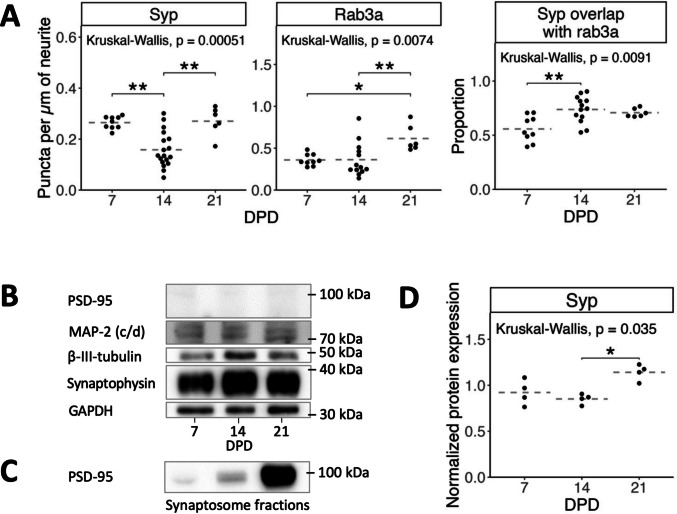
Immunofluorescence colocalization analysis and protein expression of NGF-differentiated PC12 cells. **A** Synaptophysin (left) and Rab3a (middle) puncta per µm of neurite (see Fig. 1D for example), and the proportion of synaptophysin puncta overlapping with Rab3a (right). Data represent puncta counts per non-overlapping field of view normalized by neurite length for 3 independent differentiations (2 for DPD 21), dashed lines indicate the mean. **B** Western blot of synaptophysin, MAP-2, and PSD-95 (no expression detected) and loading controls β-III-tubulin and GAPDH. **C** Western blot positive control of Anti-PSD-95 antibody (host: rabbit) applied to different rat synaptosome fractions from ref. [56]. **D** Quantification of synaptophysin expression relative to loading control (each normalized to expression at DPD 7) shows a minimal increase at DPD 21. *N* = 4 measurements from 2 independent differentiations. Statistics in **B** and **D**: Global Kruskal–Wallis test was supplemented by post hoc pairwise comparisons using Dunn’s test with Benjamini–Hochberg correction for multiple comparisons. Abbreviations: MAP-2, microtubule-associated protein 2; PSD-95, postsynaptic density protein 95; syp, synaptophysin; bTub, β-III-tubulin. *p*-values: *, < 0.05; **, < 0.01

PC12 cells were cultured and differentiated based on established protocols [4, 30, 47]. Common PC12 differentiation methods have been reviewed elsewhere [17]. Experiments were performed at day post differentiation (DPD) 7, 14, and 21. The cells showed rapid neurite sprouting, consistent with previous findings [1]. Neurite length increased until DPD 14, with no further growth observed at DPD 21 (Fig. 1B). Synaptophysin, an SV-associated protein involved in SV endocytosis and found in PC12 cell SVLVs but hardly in LDCVs [47–50], was present in a punctate pattern in comparable amounts across all observed time points, with a slight decrease in puncta density at DPD 14 and a minimally increased total expression at DPD 21 (Figs. 1A, Fig. 2B/D).

Notably, the postsynaptic marker PSD-95 [51] was not detected in either western blotting or immunofluorescence, using two well-tested antibodies (Table S1, see also positive control in Fig. 2C). This aligns with previous observations of absent postsynaptic densities in PC12 cells [22, 31], suggesting a lack of complete synaptogenesis. Conversely, Rab3a, which is associated with SVs, neuroendocrine microvesicles, and LDCVs [52–54], was variably present at different DPD (Fig. 2A) with a slight increase at DPD 21. Rab3a puncta overlapped with synaptophysin in 67 ± 13% of synaptophysin puncta over all DPD with a slight increase after DPD 7 (Figs. 1D/Fig. 2A). This suggests the development of some presynaptic-like structural organization [55]. Further characterization revealed the expression of other important presynaptic proteins in all observed cells (Fig. 1C). Synapsin I, implicated in SV handling [56], was expressed in a slightly speckled pattern concentrating at neurite swellings and branching areas without clearly coalescing into distinct puncta. Ca_V_2.2, an N-type calcium channel involved in presynaptic voltage-gated calcium influx necessary for SV release, has been shown to mediate most of the calcium influx in NGF-differentiated PC12 cells [57]. We observed Ca_V_2.2 in all cells, showing a pattern similar to synapsin I, with a stronger tendency to coalesce and occasional puncta. SV2, a protein linked to several presynaptic functions and ultrastructurally found at PC12 SVLV clusters and LDCV [47] showed a distinct punctate pattern and some overlap with synaptophysin.

### Axon- and Dendrite-Like Features in Differentiated PC12 Neurites

Although these presynaptic-like features suggest a more axonal identity of PC12 cell neurites, MAP-2, a dendrite marker [58], showed a homogenous distribution in all neurites. β-III-tubulin, a marker of neuritogenesis and neuronal axonodendritic and perinuclear compartments [59], similarly showed a uniform distribution. Both MAP-2 and β-III-tubulin displayed no notable expression trends over DPD 7–21, while β-III-tubulin still displayed 57% (*n* = 2, data not shown) expression in undifferentiated PC12 cells relative to DPD 7, likely a corollary to the known β-III-tubulin expression in pheochromocytoma [59].

**Fig. 3:**
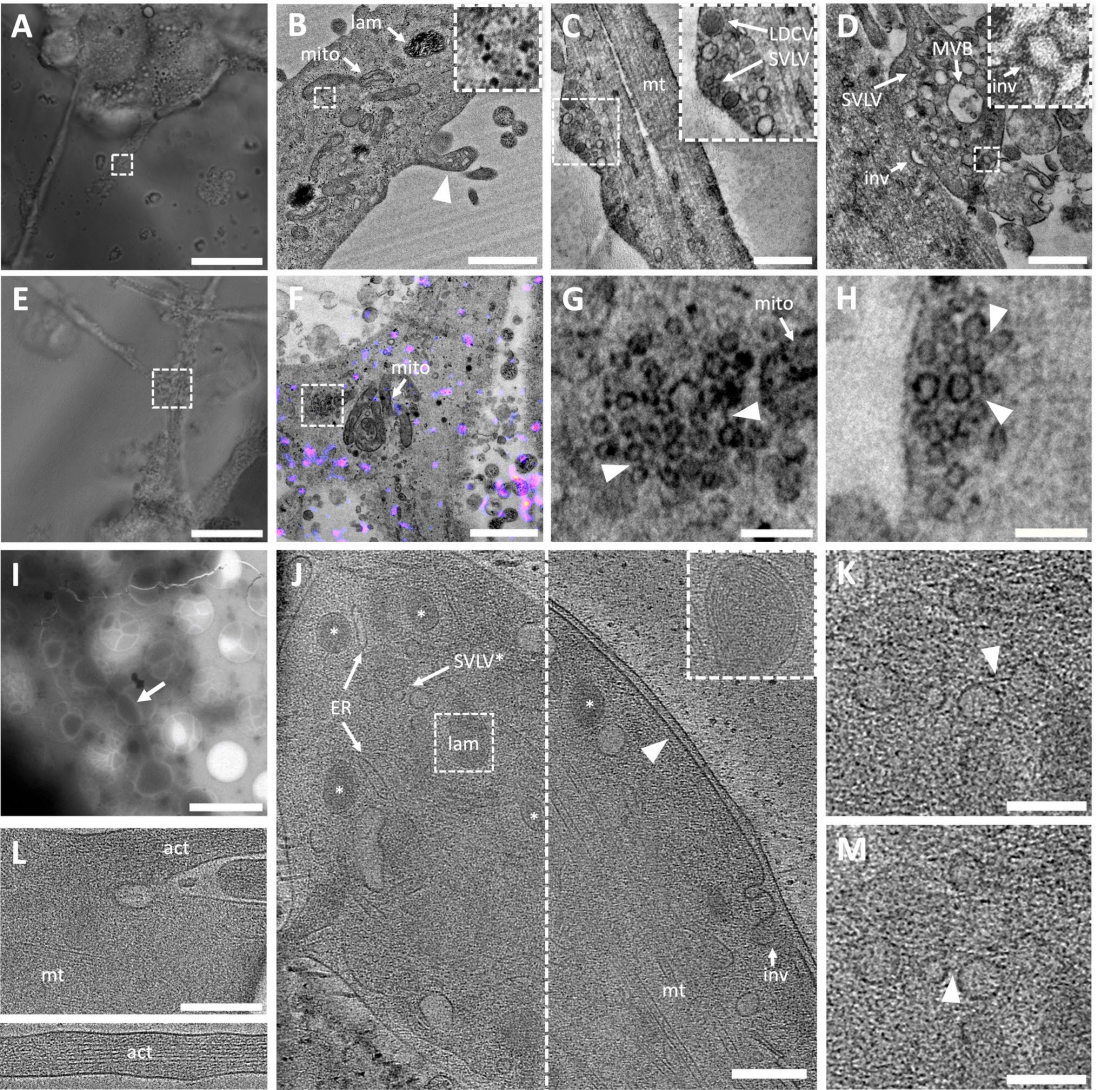
Neuron-like characteristics of PC12 cell neurites as observed in brightfield microscopy (**A**,** E**), conventional EM (**B**-**D**,** F–H**), and Cryo-ET (**I-M**, see also Supplemental Movie 1) at DPD 14 (**D-M**) and 21 (**A-C**). **A** and **B** (detail framed in **A**) Correlative light and electron microscopy images showing a dendrite-like neurite with a filopodium (arrowhead) and ribosomes (inset), **C** Axon-like neurite with tight microtubule scaffold and varicosities containing several vesicles (inset). **D** Bouton-like structure forming a tight contact with a neurite, lacking active zone characteristics and a postsynaptic density. A membrane invagination with coat is visible on the right (inset). **E** and** F** (detail framed in E)**:** Correlative light and electron microscopy images showing an axon-like neurite branching in a typical ~ 90° fashion, with SVLV cluster lacking recycling signal (fluorescent overlay: AM4-64; for details, see results). **G** and **H** SVLVs with connectors (arrowheads; note that G is an enlarged view of the framed region in **F**). **I** Cryo-ET overview of cells grown on an EM grid. The arrow shows the region depicted in **J**. **J** Presynaptic-like compartment at two different z-heights (separated by the dashed vertical line). A close cell–cell contact without synapse characteristics is indicated (arrowhead), LDCVs are highlighted using asterisks (*), the area in the dashed frame is enlarged in the inset at a different z-height, showing a multilamellar body.** K** and **M** Details of **J**, SVLVs with connectors (arrowheads). **L** Actin-containing filopodium at a more dendritic-like neurite; the bottom image shows its continuation. Abbreviations: SVLV, synaptic vesicle-like vesicles (usually surrounded by larger non-SVLV vesicles); SVLV*, SVLV cluster; lam, multilamellar body; inv, membrane invagination; mt, microtubule; act, actin; ER, endoplasmic reticulum; MVB, multivesicular body; LDCV or *, large dense core vesicle; Scale bars: **A/E**, 20 µm; **B/F**, 1 µm; **C/D**, 500 nm; **G/H**, 150 nm; **I**, 4 µm; **J/L**, 200 nm; **K/M**, 100 nm

The uniform marker distribution makes it difficult to assign an axonal or dendritic identity to PC12 neurites. Previous research has described a lack of clear axodendritic differentiation of neurites [60], although it has been reported that some stimuli may induce it [61]. Ultrastructurally, however, two types of neurites could be discerned, one exhibiting dendrite-like features (Fig. 3A/B), and one showing axon-like characteristics (Fig. 3C). The latter was found more frequently and exhibited a uniform and narrow diameter, tightly packed microtubules, little branching, and few ribosome clusters, which are all characteristics of axons [62, 63]. A minority of neurites presented a more dendritic appearance [62, 63], characterized by a larger diameter, more loosely organized cytoskeleton, and abundant ribosomes. These dendrite-like neurites also exhibited filopodia sprouting (arrowhead in Fig. 3B, L).

### Presynaptic-Like Ultrastructure and Vesicle Clustering in PC12 Varicosities

**Fig. 4:**
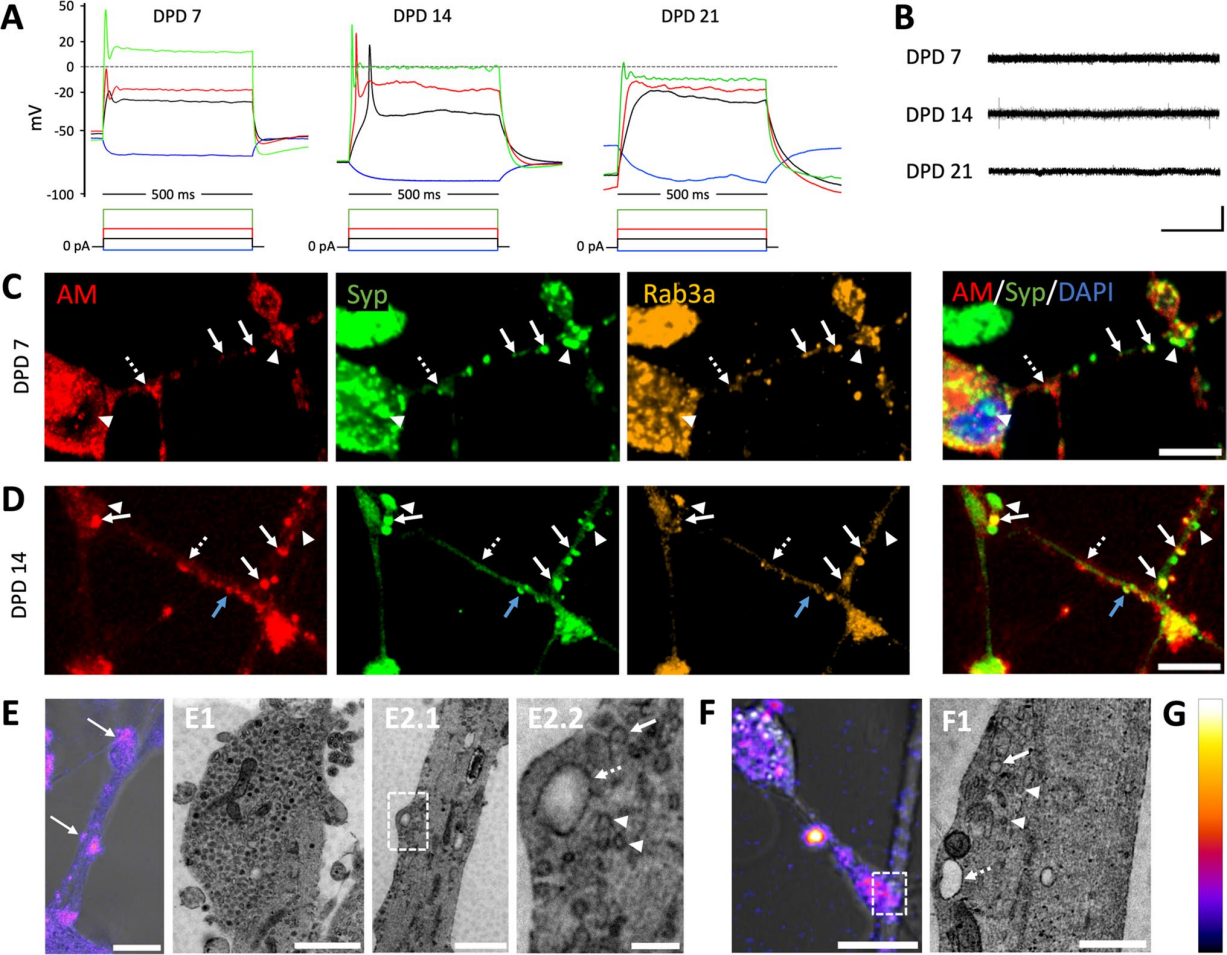
Membrane electrical characteristics and endocytosis signals. **A** Membrane potential in response to different square-shaped current pulses (blue, −20 pA; black, + 60 pA; red, + 120 pA; green, + 300 pA) in current-clamp mode. **B** Exemplary voltage-clamp recording at a fixed resting membrane potential (−60 mV) displaying a lack of spontaneous postsynaptic currents. **C** and **D** Overlap of AM4-64 recycling signal (red) and corresponding IF, i.e., synaptophysin (Syp) and Rab3a at DPD 7 and 14; the merged image on the right shows Syp, AM and DAPI (blue) overlap, the yellow color indicating Syp/AM overlap. The dashed arrow points to endocytosis without corresponding Syp signal; arrowheads indicate Syp signals without endocytosis, the regular arrows show regions of AM and Syp overlap, also overlapping with Rab3a except at the blue arrow. **E** Two loci of endocytosis (intensity-coded AM signal overlayed on brightfield image, see scale in **G**) mapped in EM to a LDCV cluster (**E2**, top arrow in E), and to a varicosity (**E2**, bottom arrow in E), in which several SVLVs (arrowheads) are found as well as vesicles larger than SVLVs (arrow, dashed arrow: considerably larger); E2.2 corresponds to the dashed square in E2.1. **F** Tight cell–cell contact (square), the corresponding electron micrograph F1 exhibiting a presynaptic-like structure on the left image half, including SVLVs (arrowheads) and vesicles larger than SVLVs (arrow; dashed arrow: considerably larger). **G** Intensity-coded color scale, starting at bit value 0 (bottom) up to 256 (top) for 8-bit images. Scale bars: **B**, horizontal 1 s, vertical 100 pA; **C–F** (fluorescent images), 10 µm; **E1, E2.1**, 1 µm; **E2.2**, 200 nm; **F1**, 500 nm

To assess how closely the observed presynaptic-like structures resemble those in neurons, we focused our analysis on axon-like neurites. These exhibited varicosities enriched with SVLVs, mitochondria, vesicles larger than SVLVs, and multivesicular bodies (MVB) (Fig. 3C/D, Fig. S1). However, most observed varicosities did not show highly organized clusters of homogeneous SVLVs as those found in bona fide presynaptic compartments [56, 64]. Smaller clusters of uniformly sized SVLVs were nonetheless observed (Fig. 3F-H), showing features of SV clusters, such as inter-vesicular filaments resembling connectors [65], which could also be observed at smaller SVLV clusters in cryo-ET (Fig. 3J/K/M, Supplemental Movie 1 of the tomogram). Connectors are thought to involve synapsin I, which we also detected (Fig. 1C, Fig. S2), although their exact composition remains unclear [56]. These clusters, however, were not found at structures resembling an active zone. The latter was also absent at tight contacts between bouton-like structures and other neurites (Fig. 3D). In contrast to cultured neurons, an abundance of LDCVs reflects the PC12 cells’ neuroendocrine origin (Figs. 3B, 4E).

Differences could be observed during the differentiation longer than 7 DPD, although most ultrastructural characteristics remained similar. The axon-like processes showed an increasing number and size of varicosities over time, with a more abundant and diverse presence of MVBs, LDCVs, and SVLVs. These features were rarely observed at DPD 7. In addition, highly organized SVLV clusters were only observed at DPD 14 and 21. However, little difference was noted regarding cell–cell contacts across the different time points.

Growth cones, the sites of neurite elongation, were observed at all examined DPD (Fig. S3A/B). Similar to growth cones of cultured neurons [66–68], they contained numerous large endosomes and SVLVs. However, they were smaller, lacked large ribosome clusters, and showed an abundance of LDCVs.

### Electrical Excitability and Lack of Postsynaptic Activity

To assess the electrical excitability of NGF-differentiated PC12 cells, whole-cell patch-clamp recordings were performed. The cells exhibited a resting membrane potential of −55 ± 16 mV across all DPD (*n* = 23, two independent differentiations; Fig. S4A), consistent with previous reports [69]. Upon depolarizing current injection, most cells generated single action potentials but no repetitive firing (Fig. 4A), indicating an immature membrane ion channel profile compared with neurons [70]. The input resistance averaged 695 ± 118 MΩ but varied widely (range 75–2,225), reflecting heterogeneous maturation (Fig. S4B).

TTX had no discernible effect on action potential morphology, whereas lidocaine markedly broadened the spike, reduced its amplitude, and diminished the subsequent afterhyperpolarization (Fig. S5A/B). These findings are consistent with the presence of TTX-resistant sodium channels in NGF-differentiated PC12 cells [71] and a partial suppression of voltage-gated and leak potassium currents. Lidocaine also altered the post-hyperpolarization sag during negative current injections, implicating involvement of hyperpolarization-activated cyclic-nucleotide-gated (HCN) channels; both mechanisms are considered in the “Discussion” section.

No spontaneous postsynaptic currents were detected under voltage-clamp conditions, indicating the absence of synaptic transmission onto the recorded cells (Fig. 4B; *n* = 8 for all DPD, two independent differentiations).

### Endocytosis Activity Mapped to Different Structures

The assessment of SV dynamics, particularly in vesicle clusters, is essential for determining the neuronal characteristics of a cell. A key feature of functional neuronal SVs is their ability to undergo swift, spatiotemporally tightly coupled, and sustained recycling—a process critical for replenishing SV pools and in sustaining synaptic transmission [72]. This recycling is constitutive and intensifies markedly upon depolarization [73].

We used the fluorescent dye AM4-64 to assess the neuron-like functionality of SVLVs in PC12 cells. The dye’s amphiphilic nature enables it to reversibly partition into the outer leaflet of membranes, thereby enhancing its fluorescence. It labels sites of endocytosis, such as synapses in neurons [74]. Adapting protocols used for primary cultured neurons [33–35], we exposed PC12 cells to a high-potassium solution with AM4-64 to trigger exocytosis and compensatory endocytosis via depolarization. To validate the effectiveness of the high-potassium stimulus, we performed representative membrane potential measurements (Fig. S5C), confirming depolarization into a voltage range (above –20 mV) that has been shown to permit calcium influx through N-type channels specifically in NGF-differentiated PC12 cells [57]—channels known to play a key role in SV recycling and neurotransmission [75]. After a variable dye incubation period to allow for endocytosis and dye uptake, the non-internalized dye was washed away and quenched, and the cells were fixed. This approach aimed to identify functional presynaptic-like structures and potential synapse precursors.

To assess the nature of these structures, we conducted correlative immunofluorescence as well as CLEM. At 15 min staining post-stimulation, AM4-64 endocytosis signals colocalized with synaptophysin and Rab3a (Fig. 4C/D). On average, 32 ± 13% of AM4-64 puncta overlapped with synaptophysin (*n* = 5 experiments at DPD 7/14 of two independent differentiations). Of the synaptophysin-AM4-64 overlaps, 40 ± 7% also colocalized with Rab3a (*n* = 3 of these experiments were stained accordingly).

In CLEM, fluorescently labeled areas of interest were first imaged by light microscopy, then processed for electron microscopy to map fluorescent signals to their corresponding ultrastructural features. Signals detected 5–15 min post-stimulation were correlated with areas in which SVLVs were surrounded by larger vesicles (Fig. 4E/F), and with lysosomes at the cell body. Perinuclear signals detected 30–60 min after stimulation were associated with the Golgi apparatus. After 24 h, several distinct puncta were observed perinuclearly and along neurites, some of which corresponded to endolysosomal structures in EM (Fig. S3C-G). Some growth cones also exhibited endocytosis signals, while some did not (Fig. S3A/B), reflecting their complex array of vesicle types and dynamics [76]. Notably, endocytosis was detected at varicosities along neurites, structures often associated with early synaptogenesis [77]. Varicosities containing loosely organized SVLVs surrounded by larger vesicles (as in Fig. 4E) exhibited endocytosis signals, while the tightly organized clusters of monomorphic SVLVs (as in Fig. 3F–H) did not. This indicates that no recent endocytosis took place in these SVLV clusters, which structurally resemble SV clusters.

An experiment using cationized ferritin as an endocytosis label for EM (as in ref. [36]) showed a comparable pattern: vesicles considerably larger than SVLVs were labeled 5 min post-stimulation, while vesicles slightly larger than SVLVs were labeled between 15 and 60 min (Fig. S3H-J). In an unstimulated sample otherwise stained like the 15-min sample, almost no ferritin labeling was detected, suggesting little basal recycling activity.

### LDCV Morphology and Stimulation Responsiveness

**Fig. 5:**
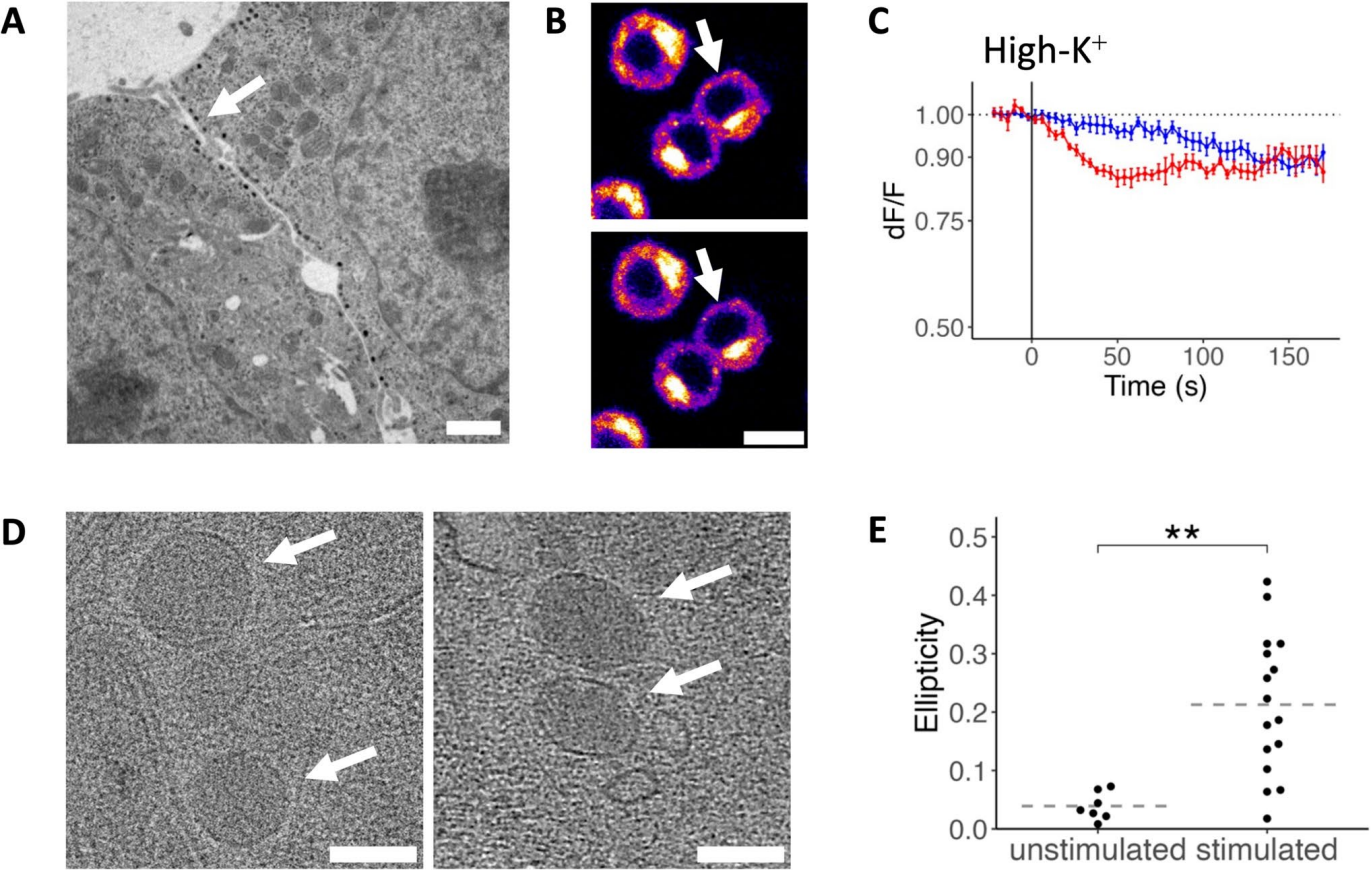
Destaining and LDCV reactivity to stimulation. **A** Electron micrograph of undifferentiated PC12 cells, showing wider cell–cell contacts than differentiated cells, and a seam of LDCVs (dark spots indicated by arrow) and SVLVs, at which destaining is observed. **B** Fluorescence imaging of undifferentiated AM4-64 stained PC12 cells just before (top) and 60 s after (bottom) high-potassium stimulation (destaining). Note the cell seams exhibiting signal loss (e.g., at arrows; intensity-coded, scale see Fig. 4G). **C** PC12 cell destaining was analyzed as the decrease in AM4-64 fluorescence over time, normalized to baseline fluorescence. Data are presented as mean values (dots) with 95% confidence intervals (error bars), corrected for photobleaching. NGF-differentiated cells (blue) showed no discernible response to high-potassium stimulation (*n* = 30 puncta from 6 independent differentiations over all DPD). However, the overall fluorescence decrease exceeded photobleaching levels, suggesting some degree of vesicle exocytosis. Undifferentiated cells (red) exhibited an accelerated destaining rate upon stimulation (selection of *n* = 4 cell seams of one differentiation). To quantify depolarization reactivity, the change in fluorescence decay slopes before and after KCl addition was assessed using a piecewise linear mixed-effects model (see “Methods”). This analysis revealed no significant difference in slope for differentiated cells (*p* = 0.531), whereas a small selection of undifferentiated cells showed a significant acceleration in decay (*p* = 0.0001). **D** Cryo-ET data showing LDCVs (arrows) flattening into an ellipse-like shape in the XY plane upon high-potassium depolarization (right), compared to the unstimulated sample (left). **E** Difference of LDCV ellipticity in unstimulated vs. stimulated cells. Mean (dashed line) and single measurements (points), *n* = 7/16 LDCVs for unstimulated/stimulated cells. Statistics: Wilcoxon rank sum test, *p* = 0.0024, indicated by two asterisks (**). Scale bars: **A**, 1 µm; **B**, 10 µm, **D**, 100 nm. *p*-values: **, < 0.01

In undifferentiated PC12 cells, LDCVs are concentrated at the cell body periphery, and the few surrounding SVLVs do not form clusters (Fig. 5A). Upon NGF differentiation, LDCVs localize to neurites, clustering in some regions (Fig. 4E) and exhibiting a more diffuse distribution at the cell body. Interestingly, we observed significant flattening of LDCVs following stimulation in NGF-differentiated PC12 cells. This reaction seems to be preserved during NGF differentiation, as it was previously described in undifferentiated PC12 cells [3]. Flattening, quantified as ellipticity, was 0.21 ± 0.12 for stimulated cells and 0.039 ± 0.024 for unstimulated cells (Fig. 5D/E).

Areas with abundant LDCVs in EM showed correlation with endocytosis signals in both undifferentiated and NGF-differentiated PC12 cells. Since LDCVs form at the Golgi apparatus rather than at exocytosis sites [78], this signal may originate from surrounding recycling vesicles of a different type or result from dye uptake during kiss-and-run exocytosis [79, 80].

### Lack of Exocytosis in Recently Recycled Vesicles

To evaluate whether the recently endocytosed structures can undergo exocytosis—a feature indicative of SV-like recycling function—we conducted destaining experiments on live cells loaded with dye following stimulation-induced endocytosis (as in ref. [81] and [35]). In high-potassium, dye-free solution, the edges of some undifferentiated PC12 cells showed a reactive fluorescence decrease likely corresponding to exocytosis of previously stained vesicles (Fig. 5B/C). It must be noted that only destaining areas were included in the analysis, and several undifferentiated cells did not show discernible reactive destaining. Conversely, in NGF-differentiated PC12 cells, no AM4-64 puncta exhibited destaining upon high-potassium stimulation, either on neurites or at cell bodies (*n* = 88 cells from two independent differentiations per DPD, two replicates each; Fig. 5C). Puncta in differentiated cells showed a near-linear decrease in fluorescence, approximating the signal of the reactive undifferentiated cells after circa 2 min. This suggests that in NGF-differentiated PC12 cells, recently endocytosed structures lack the capacity for swift exocytosis as a reaction to stimulation, thus lacking the tight spatiotemporal coupling necessary for sustained synapse vesicle-like recycling. The observed fluorescence decrease still exceeded the decrease expected by pure photobleaching, indicating the presence of constitutive recycling.

### Intercellular Connections Lack Synaptic Features

In line with previous research, our study found no fully formed synapses in NGF-differentiated PC12 cells. However, we observed several features reminiscent of synaptic junctions which were absent in undifferentiated PC12 cells. NGF-differentiation led to tight cell–cell contacts (e.g., compare Figs. 4F and), aligning with previous findings [31]. Additionally, at inter-neurite connections, some characteristics of presynaptic organization could be observed: On one side of the connection, we found an enrichment of organelles, particularly SVLVs, mitochondria, smooth ER, and multivesicular bodies, along with an endocytosis signal (Fig. 4F). Key synaptic elements, however, were absent. At presynaptic-like sites with an endocytosis signal, no clustering of monomorphic SVLVs akin to SVs was found. Additionally, both a synaptic cleft and a postsynaptic density were lacking, consistent with the absence of PSD-95.

## Discussion

### Structural and Presynaptic Features of NGF-Differentiated PC12 Cells

In our study, we examined the neuron-like properties of NGF-differentiated PC12 cells over extended culture periods (up to 21 days) to evaluate their suitability as a neuronal cell model and in particular for the study of synaptic processes. While these cells exhibit characteristics akin to primary cultured neurons, they fall short in several critical developmental aspects. The strength of our study lies in its multimodal design: by integrating ultrastructural imaging and live functional assays, we provide a more definitive evaluation of presynaptic features than previously available. Importantly, the phenotypic and molecular features we observed are highly consistent with earlier studies of NGF-differentiated PC12 cells [22, 82, 83]. These similarities lend confidence that our PC12 line faithfully recapitulates the canonical differentiation features historically attributed to this model.

PC12 cell neurites develop similarly to those of neurons, extending rapidly and containing key features such as microtubules, β-III-tubulin, MAP2, relevant organelles, and tau-1 [31]. Ultrastructural analysis by EM identified two distinct groups of neurites, resembling axons and dendrites, despite uniform dendritic MAP2 staining across neurites. This neurite growth is accompanied by growth cones and actin-driven filopodia, with only minor differences from neuron growth cones.

We demonstrate that NGF differentiation enables PC12 cells to develop presynaptic-like structures that increase in complexity with differentiation time, resembling actual presynaptic terminals. The key presynaptic proteins synaptophysin and Rab3a were found to cluster at neurites. Further, synapsin I, SV2, and Ca_V_2.2 were expressed, as has previously been shown [22, 31, 47]; notably, cholesterol-dependent syntaxin clusters were found as preferential docking and exocytosis sites in undifferentiated PC12 cells [84]. Ultrastructurally, vesicle and organelle clustering increased with prolonged differentiation, approaching architectures found at branching axons and presynaptic sites of nascent synapses in cultured neurons [29, 85]. However, we did not observe synaptic clefts or postsynaptic densities. This absence is further supported by the absence of PSD-95 immunoreactivity, consistent with previous reports [86]. Moreover, no spontaneous postsynaptic events were detected in electrophysiology, arguing not only against synaptic specializations but also against detectable signals that might arise from extrasynaptic transmission—as would be expected in more central, cholinergic-type signaling [87].

Given their chromaffin origin and NGF-induced cholinergic phenotype [19], future work may more specifically probe postsynaptic precursors and cholinergic scaffolds. Although PSD-95 dominates glutamatergic synapses, it is not the principal MAGUK at autonomic ganglionic synapses, where PSD-93 fulfills a similar anchoring role for nicotinic acetylcholine receptors [88, 89]. The anti-PSD-95 antibody used here (clone 7E3; Supplemental Table 1) cross-reacts with PSD-93 [90]. Thus, the absence of staining argues against the presence of either protein under our conditions. Cholinergic synapses generally exhibit less canonical scaffolding than glutamatergic ones yet retain distinguishable ultrastructural synaptic features [91, 92]. In the ciliary ganglion, nicotinic synapses display receptor clustering organized by neuroligin, EphB2, and variably by all four PSD-95-family MAGUKs (PSD-95, PSD-93, SAP97, SAP102) [93]. Central cholinergic synapses remain poorly defined and may integrate multiple organizing principles; recent ultrastructural studies revealed ACh/GABA co-transmitting synapses [92], suggesting that scaffolds such as gephyrin, classically inhibitory, could also delineate cholinergic postsynaptic sites.

Although dystrophins are best known for their postsynaptic role at the neuromuscular junction (NMJ)—a cholinergic synapse—they also support synaptogenesis and synaptic stability in neurons [94]. Several NMJ- and cholinergic-associated proteins have been detected in NGF-differentiated PC12 cells, indicating expression of structural components relevant to synapse formation. Among dystrophins, only Dp71 has been identified; in L6-myocyte co-cultures, it co-localizes in PC12 cells with utrophin, acetylcholine receptors, and synaptophysin, consistent with a presynaptic-like organization, possibly at autoreceptive sites [95]. Additional NMJ-related molecules include rapsyn and agrin, ACh receptor-aggregating factors, in PC12 cells so far observed only at the mRNA level [96–98]. Drebrin, a cytoskeletal organizer in NMJ and dendritic spines [99], occurs near synaptotagmin-positive compartments in NGF-differentiated PC12 cells [31] but also in growth cones [100]. Together, these observations suggest that PC12 cells express parts of the synaptogenic molecular machinery, although their organization appears primarily presynaptic rather than reflecting a complete synaptic phenotype.

Reports of synapse formation in PC12 cells co-cultured with myocytes or neurons [23, 24] indicate that these cells can engage in context-dependent structural interactions when exposed to suitable partner cells. Such findings suggest that specific environmental or transsynaptic cues may promote more advanced differentiation states than those observed under monoculture conditions. However, the evidence remains limited: one study reports indirect indications of synaptic activity at a NMJ [24] and another—providing ultrastructural evidence of a single synapse [23]—has not been replicated. To our knowledge, beyond that report, no conclusive ultrastructural demonstration of PC12 synapses exists; this includes ref. [31], which shows neither synaptic clefts nor postsynaptic densities. In our monoculture experiments, such cues likewise appear to be absent, and the resulting organization remains confined to presynaptic-like structures, prompting a functional assessment of their competence.

### SVLV Recycling Dynamics and Functional Limitations

To examine the functional basis of this apparent inhibition of synaptogenesis, we investigated the recycling competence of SVLVs, as sustained vesicle turnover is a prerequisite for the formation and maintenance of active zones during synapse development. In primary neurons, active zone formation and mature synapse development require transsynaptic communication [28]. This process is supported by SV recycling, including sustained local endo- and exocytosis, which emerges early. It was observed to appear in Xenopus spinal neurons before any sort of cell–cell contact [101] and within an hour of initial axonodendritic contact in hippocampal culture [29].

Both undifferentiated and differentiated PC12 cells are widely used as models for neurosecretion and SNARE protein interactions due to their coordinated exocytosis machinery [2, 4, 6, 11, 102]. High-potassium stimulation during AM4-64 dye incubation revealed numerous endocytosis sites in neurites and cell bodies of NGF-differentiated PC12 cells. These likely correspond to sites of prior exocytosis, consistent with the principle of membrane homeostasis described in neuroendocrine cells [103] and neurons [104]. Approximately one-third of these sites colocalized with synaptophysin in retrospective immunofluorescence, and 40% of these also colocalized with Rab3a, indicative of loci containing SVLVs and/or LDCVs. CLEM analysis mapped these endocytosis signals to specific subcellular ultrastructural features, including varicosities and presynaptic-like structures at cell–cell contacts. Other than LDCVs (discussed below), these contained clusters of loosely organized SVLVs and larger vesicles resembling early/recycling endosomes [105–107], which are known precursors to PC12 SVLVs [108]. Previous research on undifferentiated PC12 cells suggests that both endosomes and SVLVs (via small fusion pores during kiss-and-run exocytosis) are stained [106, 109]. Indeed, in SVLVs of undifferentiated PC12 cells, kiss-and-run is thought to be the most common type of evoked exo-/endocytosis [106, 110, 111]. Accordingly, a ferritin staining experiment showed labeling in endosomes but not in SVLVs, consistent with the notion that ferritin is too large to pass through the small fusion pore formed during kiss-and-run exocytosis.

In differentiated PC12 cells, recently endocytosed structures failed to undergo exocytosis upon repeat stimulation, as demonstrated by AM4-64 destaining experiments. These indicated only constitutive recycling, without evidence of stimulus-evoked release (Fig. 5C). In contrast, undifferentiated PC12 cells displayed inconsistent destaining behavior: some responded to repeat stimulation with reactive destaining (as in ref. [112] and in indirect observations in ref. [15, 16]), while many did not (as in ref. [106]). For differentiated PC12 cells, the lack of reactive destaining suggests incomplete recycling of SVLVs to exocytosis-competent states within the 15-min staining period. Earlier studies using endocytosis labels predominantly applied them without stimulation, thereby tracking de novo SVLV formation via endosomes [19, 113–115]. This process parallels SV biogenesis in neurons, where membrane components and synaptic proteins are recycled through plasma membranes, endosomes, and mature SVLVs or SVs [72, 116]. Our data suggest a divergence in SVLV behavior post-exocytosis compared to SVs, potentially due to an intermediate step in SVLV recycling that temporally or spatially uncouples endocytosis and re-exocytosis. Alternatively, different SVLV populations might be utilized during subsequent stimulations. Given the strong 2-min high-potassium stimulus, the latter explanation seems less likely. Previous studies have shown that SVLV clusters become depleted and neurotransmitter release returns to a basal rate after 5 min of sustained high-potassium stimulation (albeit without differentiating SVLV and LDCV release) [22, 117].

Incomplete recovery of N-type calcium channels (Ca_V_2.2) due to insufficient hyperpolarization following dye-loading depolarization could, in principle, contribute to the absence of AM4-64 destaining. However, several factors argue against this explanation. N-type calcium channels have previously been shown to recover from fast and slow inactivation at –60 mV [118]. In our experiments, cells were given a 15-min recovery period after stimulation, allowing ample time for reactivation of a substantial fraction of channels. Moreover, the average resting membrane potential across recorded cells was –55 mV, with a broad distribution including many cells at more hyperpolarized levels (Fig. S4). Given this distribution and the total number of cells analyzed (*n* = 88), it is unlikely that the uniformly absent destaining response resulted solely from insufficient recovery of calcium channel function.

Clusters of SVLVs with higher-order organization resembling SV clusters showed no signs of recent recycling, suggesting that they may parallel a reserve pool of SVs [119]. In contrast to synapses, which possess a readily releasable SV pool tethered to the plasma membrane [119], PC12 cells may lack a comparable SNARE-independent tethering mechanism that facilitates rapid neurotransmitter release and sustained, spatiotemporally coupled recycling. Supporting this, previous electrophysiological research has shown a latency from intracellular calcium stimulation to exocytosis of around 24 ms in SVLVs (of undifferentiated PC12 cells), compared to 0.3 ms in neuronal SVs [120]. The lack of reactive destaining suggests an absence of spatiotemporally coupled SV-like recycling, which is critical for maintaining transsynaptic communication during synaptogenesis.

### Additional Synaptogenesis Constraints and LDCV Behavior

Additional factors involved in transsynaptic signaling may also restrict the neuronal development of NGF-differentiated PC12 cells. NGF differentiation increases neuron-like characteristics related to ACh metabolism—including increased expression of ACh esterase, nicotinic ACh receptors, and choline acetyltransferase [22, 24, 121]. It increases calcium and sodium channel expression as well as calcium and TTX-sensitive and TTX-resistant sodium currents [71, 122–124]. Nonetheless, the low density of sodium channels contributes to an immature electrophysiological phenotype characterized by single, variably shaped action potentials [11, 125–127]. In our recordings, the lack of TTX effect on spike morphology indicates a predominance of TTX-resistant channels.

Lidocaine, besides its state-dependent block of sodium channels [128], suppresses several potassium conductances present in NGF-differentiated PC12 cells, including delayed-rectifier, inward-rectifier, and two-pore-domain (TASK/TREK) leak currents [129–135]. Its broad spectrum thus contrasts with the selective action of TTX and explains the pronounced effects observed on spike morphology and membrane responses. Under these conditions, a sag (rebound) was evident at strong hyperpolarization (Fig. S5B), consistent with partial activation of an HCN-mediated current unmasked by reduced potassium leak [136]. The observed slow activation kinetics match the properties reported for HCN2, which—along with HCN4—is upregulated by NGF in PC12 cells [126]. At smaller hyperpolarizations, the lack of sag may reflect stronger HCN inhibition by lidocaine at less negative voltages [136]. Overall, the broad variability in resting potential and input resistance, together with the weak TTX sensitivity and the susceptibility to lidocaine, indicates electrophysiological immaturity and heterogeneity in ion-channel profiles compared with mature neurons. Future voltage-clamp studies using selective blockers could further resolve the ionic mechanisms underlying their limited excitability.

In addition to the above synaptogenic limitations, we examined LDCV dynamics. LDCVs in undifferentiated PC12 cells have been reported to undergo both kiss-and-run exocytosis with rapid, localized re-endocytosis [137, 138], and spatiotemporally uncoupled exo- and endocytosis [139]. Both of these are also observed in neuronal dense-core vesicle handling [78, 79]. However, it is important to note that in live destaining experiments, fluorescence signals from SVLVs, endosomal compartments, and LDCVs cannot be reliably distinguished. Notably, none of these sites exhibited stimulation-evoked destaining. This aligns with expectations, as even LDCVs retrieved via local kiss-and-run are reported to enter a refractory state and are not immediately reused [137]. In undifferentiated PC12 cells, LDCVs have also been observed to undergo elliptical distortion in response to cytosolic calcium-based stimulation [3]. While the functional significance of this deformation remains unclear, we similarly observed this response in NGF-differentiated PC12 cells upon high-potassium induced depolarization. Further studies may clarify whether this mechanism is also present in neurons.

In summary, NGF-differentiated PC12 cells develop several neuron-like characteristics, including robust neurite outgrowth, axon-dendrite differentiation, partial electrical excitability, and presynaptic-like protein clustering and ultrastructures. However, they lack the membrane channel composition typically associated with mature neuronal excitability. Moreover, the absence of sustainable, spatiotemporally coupled SVLV recycling may limit the transsynaptic signaling processes necessary for synaptogenesis. These findings suggest that such coupling may represent a late feature of neuronal differentiation—as observed in iPSC-derived neurons [140]—that is not recapitulated in this model. As a result, NGF-differentiated PC12 cells are limited in their utility for studying synaptogenesis or mature neuronal synaptic functions. Nonetheless, they remain a valuable tool for investigating regulated exocytosis, dense-core vesicle biology, and the upstream molecular events of neuronal polarization as well as neurite and presynaptic assembly.

## Conclusion

Our multimodal analysis confirms that NGF-differentiated PC12 cells recapitulate several neuronal features, including neurite outgrowth and aspects of presynaptic-like organization. Rigorous ultrastructural assessment reinforces their morphological resemblance to neurons. However, key aspects of synaptic function are absent—most notably, defined active zones and sustained synaptic vesicle dynamics, as shown by EM, CLEM, and functional assays. These limitations render PC12 cells unsuitable for investigating synaptogenesis or neuron-like synaptic transmission. By precisely delineating the boundaries of this widely used model, our findings offer clarity for future methodological choices in synaptic research. Further studies may explore whether targeted differentiation protocols or co-culture systems could overcome these constraints. Ultimately, this work highlights the importance of aligning model systems with the specific cellular processes under investigation.

## Supplementary Information

Below is the link to the electronic supplementary material.

ESM1 (DOCX 18.5 MB)

ESM2 (MP4 19.0 MB)

## Data Availability

Data supporting the findings of this study are available within the paper and supplementary materials. The raw data is available from the corresponding author upon reasonable request.
