# Supplementary material for: Assessing the Potential of NGF-Differentiated PC12 Cells as a Model for Synaptic Transmission
Source: Mol Neurobiol. 2025 Dec 10;63(1):281. doi: 10.1007/s12035-025-05562-5 (PMC12696136; doi:10.1007/s12035-025-05562-5)
Supplement: Supplementary file 1 — (DOCX 18.5 MB) [file 12035_2025_5562_MOESM1_ESM.docx]

Assessing the Potential of NGF-Differentiated PC12 Cells as a Model for Synaptic Transmission
–Supplemental Materials

**Grischa Ott^1^**, ORCID: 0009-0006-0974-7321, grischa.ott@students.unibe.ch; **Jana Leuenberger^1^**, jana.leuenberger@unibe.ch; **Niels Ntamati^2^**, niels.ntamati@gmail.com; **Andrey Ivanov^1,3^**, aivanov@medfac.mu-sofia.bg; **Thomas Nevian^2^**, thomas.nevian@unibe.ch; **Iman Rostami^1,$^**, ORCID: 0000-0002-1344-0748 iman.rostami2@unibe.ch; **Benoît Zuber^1,$^**, ORCID: 0000-0001-7725-5579, benoit.zuber@unibe.ch
**1**: Institute of Anatomy, University of Bern, 3012 Bern, Switzerland
**2**: Department of Physiology, University of Bern, 3012 Bern, Switzerland
**3**: Department of Anatomy, Histology, and Embryology, Medical University-Sofia, 1431 Sofia, Bulgaria
**$**: Corresponding authors
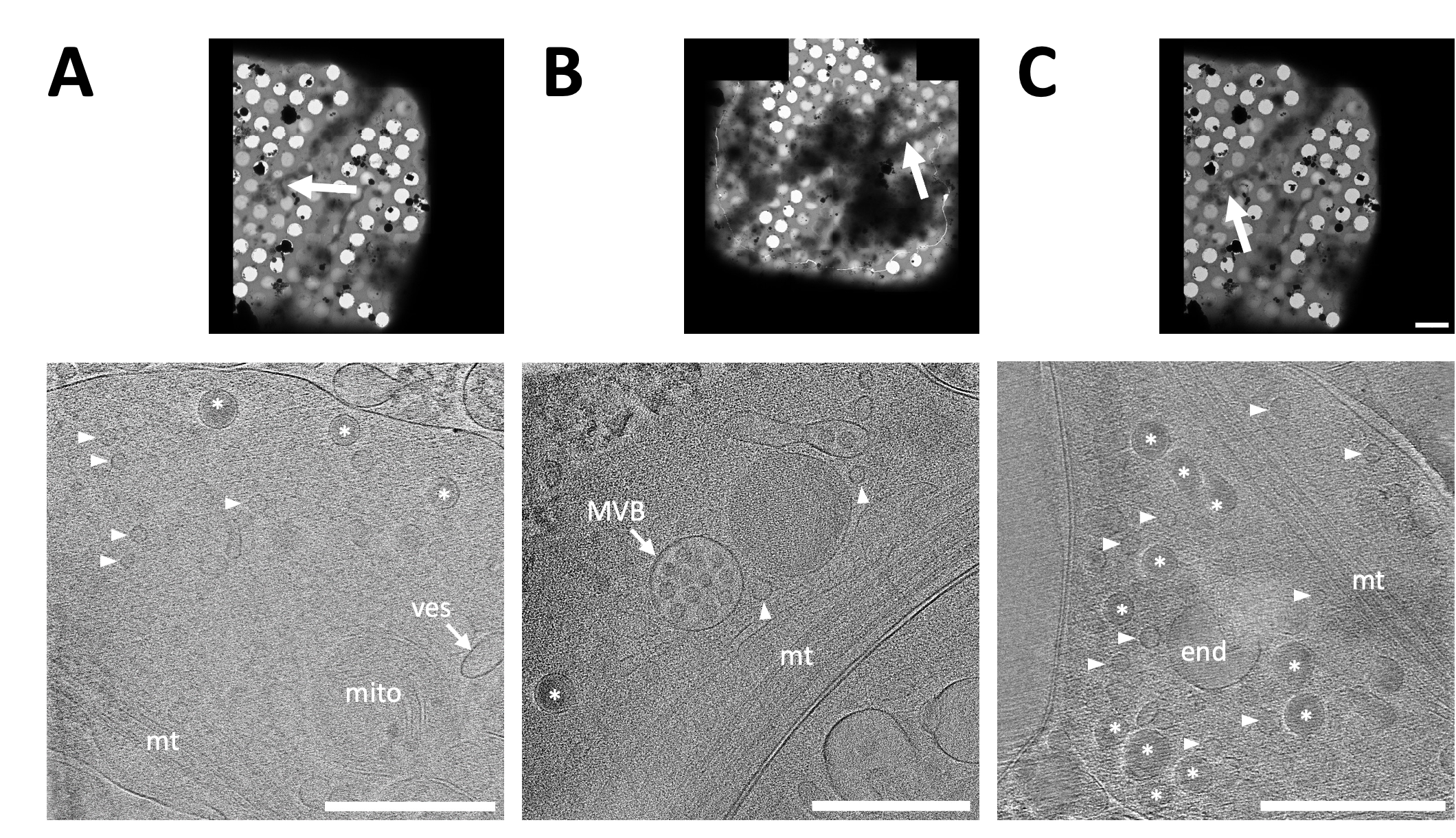


**Fig. S1** **A-C**: Additional tomograms showing organelle clustering and loci of small SVLVs (arrowheads) clusters at DPD 21, LDCVs are indicated by asterisks (*). Top: Search map (overview micrograph), bottom: tomogram at arrow in search map. In **C**, a moon-shaped infolding may correspond to an endosome (end), specifically, a MVB precursor. **Abbreviations**: SVLV, synaptic vesicle-like vesicles; mt, microtubule; MVB, multivesicular body; mito, mitochondria; end, endosome. **Scale bars:** 500 nm

**Fig. S2** Synapsin I expression of PC12 cells assessed using Western blot in the undifferentiated state (UD) and at NGF-differentiated at DPD 7, 14, 21.

**Fig. S3** Supplemental images of endocytosis signals stained with AM4-64 (**A-G**; intensity-coded, scale see Fig. 3G) or labelled using cationized ferritin (**H-J**, exemplary particles indicated by arrowheads).
**A**: Example of growth cone (DPD 7) with overlayed AM4-64 signal indicating recent endocytosis. **B**: Example of growth cone (DPD 14) without signal of recent endocytosis. **A1/B1**: Electron micrographs of the regions outlined in **A** and **B**, respectively. **C-G**: AM4-64 at different timepoints post-staining (separate experiments). At 5 min, few peripheral and perinuclear puncta appear; at 15 min both increase. By 30-60 min, puncta converge perinuclearly, and after 24 h, the endocytosis signal is distributed in distinct puncta both at the perikaryon and the neurites. **D1**: Correlative electron micrograph of square in B (at 15 min) relating the endocytosis signal to an endolysosomal structure. **D2**: Detail of dashed square in **D1**. **F1**: Correlative electron micrograph of square in F (at 60 min), showing the Golgi apparatus as a correlate for the endocytosis signal. **F2:** Detail of dashed square in F1. **G1**: Correlative electron micrograph of G (24 h), showing the signal associated with endolysosomal structures. **G2/3**: Details of solid/dashed squares in G1, highlighting multilamellar bodies. **H**: At 5 min of post-stimulation, ferritin is localized in large vesicles but not in SVLVs. **I**: At 30 min, ferritin is found in vesicles only slightly larger than SVLVs (~75 nm in diameter). **J1**: At 30 min, ferritin remains absent in SVLVs, shown at a varicosity in a small cluster. **J2**: Detail of J1 (square) showing SVLVs and a tubular structure resembling the endoplasmatic reticulum. **Abbreviations**: ER?, putative endoplasmic reticulum, ves, vesicles larger than SVLV; ribo, ribosome cluster; fil, filopodia. **Scale bars**: **A**/**B**, 10 µm; **C**-**G**, 20 µm; **A1**/**D1**, 1 µm; **B1**/**D2**/**F2**/**G2**/**G3**, 500 nm; **F1**/**G1**, 5 µm; **H**, 200 nm; **I**/**J2**, 100 nm; **J1**, 300 nm

**Fig. S4** Membrane resting potentials (**A**) and input resistance (**B**) of PC12 cells at different DPDs show heterogeneous electrical maturity. The horizontal dashed line represents the mean.

**Fig. S5** Current-clamp recordings from NGF-differentiated PC12 cells (A, B: DPD 7; C: DPD 14) reveal TTX-resistant sodium conductances and membrane depolarization in response to high-potassium stimulation. In all experiments, cells were subjected to incremental current injections from -60 pA to +320 pA in 20 pA steps. **A**: Application of tetrodotoxin (TTX, 10 µM, ~10 min) did not alter action potential morphology, supporting the predominance of TTX-resistant sodium channel activity. **B**: Lidocaine treatment (300 µM, ~10 min) broadened and delayed the single action potential spike and reduced the after-hyperpolarization, consistent with partial inhibition of voltage-gated sodium and potassium channels. During hyperpolarizing current injections, membrane responses changed markedly: at -60 pA, the instantaneous (pre-sag) voltage deflection increased from -133 mV (no lidocaine) to -211 mV (lidocaine), corresponding to an increase in instantaneous input resistance from 1.63 to 3.02 GΩ – consistent with a voltage-dependent decrease in total potassium conductance. This was accompanied by a stronger sag (46 vs. 159 mV; sag ratio 0.47 vs. 0.88). At -40 and -20 pA, this was not observed and the sag evident in lidocaine-free conditions nearly disappeared with lidocaine. At depolarizing current steps, voltage traces reached a higher steady-state level with lidocaine, indicating an additional rise in input resistance on the depolarized side. Together, these findings illustrate lidocaine’s combined effects on sodium, potassium, and HCN channel activity. **C**: Perfusion with high-potassium artificial cerebrospinal fluid (ACSF; 50 mM KCl, 77.5 mM NaCl) resulted in a substantial depolarization of the membrane potential. Upon washout and return to standard ACSF, both the resting membrane potential and evoked action potentials recovered

**Table S1** Antibodies & dyes used

| Type | Descriptor / Conjugate | Manufacturer | Product number | Host | Dilutions IF / WB |
| --- | --- | --- | --- | --- | --- |
| Primary Antibodies | Anti-Synaptophysin | Sigma-Aldrich | S5768 | Mouse monoclonal | 1:1000 / 1:5000 |
|  | Anti-Rab3a | Proteintech | 16865-1-AP | Rabbit polyclonal | 1:1000 / 1:5000 |
|  | Anti-PSD-95 (Clone C2D4) | Abcam | ab238513 | Rabbit monoclonal | 1:1000 /  1:1000-1:5000 |
|  | Anti-PSD-95 (Clone 7E3) | Invitrogen | MA5-45141 | Mouse monoclonal | 1:1000 / - |
|  | Anti-MAP2 | Invitrogen | PA1-10005 | Chicken polyclonal | 1:1000 / 1:5000 |
|  | Anti-β-III-tubulin | Novus Biologicals | NB100-1612 | Chicken polyclonal | 1:1000 / - |
|  | Anti-synapsin I | Sigma-Aldrich | S193 | Rabbit polyclonal | 1:1000 / 1:5000 |
|  | Anti-SV2 | Abcam | ab77177 | Goat polyclonal | 1:1000 / - |
|  | Anti-Ca_v_2.2 | Alomone labs | #ACC-002 | Rabbit polyclonal | 1:1000/ - |
|  | Loading control: Anti-β-tubulin / HRP | Invitrogen | MA5-16308-HRP | Mouse monoclonal | - / 1:5000 |
|  | Loading control: Anti-GAPDH / HRP | Invitrogen | MA5-15738-HRP | Mouse monoclonal | - / 1:5000 |
| Secondary Antibodies | Anti-Rabbit / Alexa-488 | Invitrogen | A11004 | Goat polyclonal | 1:1000 / - |
|  | Anti-Rabbit / Alexa-568 | Invitrogen | A11011 | Goat polyclonal | 1:1000 / - |
|  | Anti-Mouse / Alexa-568 | Invitrogen | A11004 | Goat polyclonal | 1:1000 / - |
|  | Anti-Chicken / Alexa-647 | Jackson ImmunoResearch | 703-605-155 | Donkey polyclonal | 1:1000 / - |
|  | Anti-Rabbit / HRP | Invitrogen | 31466 | Goat polyclonal | - / 1:5000 |
|  | Anti-Chicken / HRP | Thermo Fisher Scientific | A16054 | Goat polyclonal | - / 1:5000 |
| Dyes | DAPI (4',6-diamidino-2-phenylindole-dihydrochlorid) | Sigma-Aldrich | D9542 | - | 1 µg/ml |
|  | AM4-64 | Biotium | 70025 | - | 10 µM |
| Quencher | SCAS (4-Sulfonato calix[8]arene, sodium salt) | Biotium | 70037 | - | 0.5 mM |
